# Secondary infection in severe COVID-19 patients: clinical and microbial patterns at a tertiary hospital in Vietnam

**DOI:** 10.1186/s12879-025-12479-w

**Published:** 2025-12-31

**Authors:** Pham Hong Anh, Chau Vinh, Nguyen Phu Huong Lan, Ho Quang Minh, Le Thi Quynh Ngan, Huynh Phuong Thao, Mai Thu Si Nguyen, Nguyen Thanh Dung, Yeonji Jeon, Se Eun Park, Pham Thanh Duy

**Affiliations:** 1 Oxford University Clinical Research Unit, 764 Vo Van Kiet, District 5, Ho Chi Minh City, Vietnam; 2 Hospital for Tropical Diseases, Ho Chi Minh City, Vietnam; 3 International Vaccine Institute, Seoul, South Korea; 4 Yonsei University Graduate School of Public Health, Seoul, South Korea; 5 Center for Tropical Medicine and Global Health, Nuffield Department of Clinical Medicine, Oxford University, Oxford, UK

**Keywords:** Secondary infection, COVID-19, Multidrug resistance, XDR, Nosocomial infections

## Abstract

**Background:**

COVID-19 predisposes patients to secondary infection, resulting in increased mortality worldwide. It is thus crucial to identify the causes of secondary infection and their clinical outcomes to devise future prevention and control strategies. This study aimed to report the clinical and microbiological features of bacterial and fungal secondary infections in severe COVID-19 patients during the peak of the pandemic in Vietnam.

**Methods:**

We collected data from 3,789 confirmed COVID-19 patients hospitalized at the Hospital for Tropical Diseases in Ho Chi Minh City between 2020 and 2021. Demographics, infection pathogens, treatment characteristics, and patient outcomes were recorded. Univariate and multivariate analyses were performed to identify risk factors associated with mortality.

**Results:**

Microbiologically confirmed secondary infection was identified in 17.7% (651/3,682) of hospitalized COVID-19 patients. The most frequent comorbidities were cardiovascular diseases (74.9%), hypertension (65.9%), and diabetes (54.5%). The overall survival rate was 83.5% (3,075/3,682), highest in patients without secondary infection (97.2%), and dropped dramatically to 35.6% in those with microbiologically confirmed secondary infection. Out of 2,649 pathogens identified, Gram-negative bacteria accounted for 53.8% of isolates, followed by fungi (32.5%) and Gram-positive bacteria (13.7%). Notably, the predominant bacterial (*A. baumannii*, *K. pneumoniae*, *P. aeruginosa*) and fungal pathogens (*C. tropicalis*, *C. albicans*) exhibited high resistance rates to last-resort antibiotics (carbapenems, colistin) and antifungal drugs (fluconazole), respectively. Regression analyses found that secondary infection, older age, chronic kidney disease, cardiovascular disease and mechanical ventilation were the independent predictors of mortality.

**Conclusions:**

Secondary infection in COVID-19 patients was predominantly caused by highly resistant Gram-negative bacteria, and was associated with older patients who had comorbidities and underwent invasive procedures. Patients with secondary infection experienced higher mortality. Our work underscores the need for strengthening infection prevention measures and antibiotic stewardship programs to prevent nosocomial infections and better prepare for future epidemics.

## Introduction

Since the first report of COVID-19 in late 2019, the SARS-COV-2 virus has spread over the world, causing massive epidemics in many countries. As of mid-2024, there have been more than 775 million confirmed cases of COVID-19, including seven million deaths [1]. Similar to other respiratory viral diseases, COVID-19 predisposes patients to secondary infections such as bacteremia, nosocomial pneumonia, urinary tract, and skin infection, particularly in critically ill cases who need intensive care treatment [2–5]. The prevalence of secondary infection was reported as high as 24% in COVID-19 cases, which often leads to a significant increase in fatality [3, 6–8].

Vietnam experienced four waves of the COVID-19 pandemic [9], during which the country maintained a low number of cases in the first three waves. However, in April 2021, the fourth wave saw a dramatic surge, with cases reaching nearly 10,000 per day at its peak [10]. Ho Chi Minh City (HCMC) was the most heavily impacted province, with over 443,000 cases, representing 41% of the total cases in southern Vietnam [9–11]. During the peak of the pandemic between late April and September 2021, the estimated infection and death rates of COVID-19 in HCMC were 3,723 per 100,000 population and 145 per 100,000 population, respectively (https://covid19.ncsc.gov.vn/dulieu). This corresponded to a case fatality ratio of about 4.2%, which was higher than the global average of ~ 2.2% (https://www.who.int/emergencies/diseases/novel-coronavirus-2019, Accessed May 7, 2021). The dramatic surge in COVID-19 patients significantly overwhelmed the healthcare system and increased the risk of secondary infection among hospitalized patients.

Although the global emergency phase of the COVID-19 pandemic has ended, a thorough review of the causes and outcomes of secondary infection in COVID-19 patients is required for future preparedness and strategic response. In this study, we report the occurrence and clinical outcomes of bacterial and fungal secondary infections in COVID-19 patients who were hospitalized at the Hospital for Tropical Diseases (HTD) in HCMC, Vietnam, during 2020–2021.

## Materials and methods

### Study design and site

We conducted a retrospective data extraction, curation, and analysis of the hospital records of all COVID-19 patients admitted to HTD in HCMC from January 2020 to December 2021. HTD is the largest referral hospital for infectious diseases in southern Vietnam, with approximately 660 beds and receiving over 2,500 outpatients per day. The hospital has served as a referral center for severe COVID-19 cases in HCMC since the beginning of the pandemic. In June 2021, HTD was officially designated as a COVID-19 treatment facility for severe patients in response to the peak of the pandemic in HCMC. During the height of the pandemic, Vietnam implemented a three-tier COVID-19 treatment model to avoid overwhelming of the healthcare system. The first tier provides care for asymptomatic or mild patients, the second tier receives non-critical moderate-to-severe patients who require oxygen supply and pneumonia treatment, and the third tier, including HTD, is reserved for critically ill patients with severe symptoms.

### Definition of secondary infection in COVID-19 patients

In this study, ‘microbiologically confirmed secondary infection’ was defined as a positive microbiological culture for at least one clinically relevant pathogen (bacteria and/or fungi) from blood, urine, wound swab/pus, or respiratory tract samples (sputum, bronchoalveolar lavage, pleural fluid, endotracheal aspirate), that were collected after 48 h of direct admission or within seven days of transfer from another treatment facility [12]. COVID-19 patients with microbiological culture were performed after 48 h of direct admission or within seven days of transfer from another treatment facility, but no microorganisms were found, and they were grouped into ‘suspected secondary infection’. COVID-19 patients without indications for microbiological culture were classified as ‘no secondary infection’.

A new episode of secondary infection was recorded if occurring at least seven days between two consecutive microbiological isolations, including the same or a different organism. Polymicrobial secondary infection was defined as the isolation of more than one microorganism (including bacteria and fungi) from the same or different clinical specimens during an episode.

### Microbiological culture

For blood culture, two to four bottles with 8–10 mL of blood per bottle for adults and 2–5 mL for children were routinely obtained and inoculated into aerobic and anaerobic blood culture bottles, which were subsequently incubated at 35 ± 2 °C in BACT/ALERT VIRTUO (Bio-Mérieux, France) or BD BACTEC FX (Becton Dickenson, USA) automated analyzer for up to five days. Sub-culture was performed on fresh sheep blood, MacConkey, and chocolate agars when the machine indicated a positive signal. Organisms were identified by MALDI-TOF (Bruker, Germany) and Vitek 2 Compact (Bio-Mérieux, France) automated identification and antimicrobial susceptibility test (AST) systems. For blood culture, Coryneform (*Corynebacterium*, etc.), Coagulase-negative Staphylococci (CoNS), Micrococci, Propionibacterium, Bacillus, alpha-hemolytic *Streptococci*, environmental Gram-negative *Bacilli*, and non-pathogenic *Neisseria* were regarded as contaminants from blood culture, unless isolated from two or more separate blood culture sets [13].

For sputum culture, sample quality was assessed using Bartlett’s grading system [14], followed by plating onto selective media for bacterial isolation. For tracheal aspirate (TA) and urine culture, samples were quantitatively plated onto selective media, and bacterial identification and AST were performed for known pathogens from TA with colony count ≥ 10^6^ cfu/mL and uropathogens with colony count ≥ 10^5^ cfu/mL.

Multi-drug resistant (MDR), extensively-drug resistant (XDR), and pan-drug resistant (PDR) bacteria were reported for the predominant bacterial and fungal pathogens. For bacterial pathogens, MDR was defined as acquired non-susceptibility to at least one agent in three or more antimicrobial classes, XDR was defined as non-susceptibility to at least one agent in all but two or fewer antimicrobial categories, and PDR was defined as resistance to all antibiotics [15]. The following antimicrobial categories or agents were used to distinguish MDR, XDR, and PDR: *Enterobacteriales*: aminoglycosides, carbapenems, cephalosporins, cephamycins, ciprofloxacin/levofloxacin, trimethoprim-sulfamethoxazole, fosfomycin, penicillins + β-lactamase inhibitors, tetracyclines. *Pseudomonas aeruginosa*: aminoglycosides, carbapenems, cephalosporins, fluoroquinolones, polymyxins, penicillins + β-lactamase inhibitors. *Acinetobacter spp.*: aminoglycosides, carbapenems, fluoroquinolones, extended-spectrum cephalosporins, trimethoprim-sulfamethoxazole, tetracyclines, polymyxins, penicillins + β-lactamase inhibitors. *Staphylococcus aureus*: gentamicin, fluoroquinolones, glycopeptides, tetracyclines, ansamycins, trimethoprim-sulfamethoxazole, tigecycline, clindamycin, daptomycin, linezolid, fosfomycin, oxacillin, macrolides/lincosamides. *Enterococcus spp.*: fluoroquinolones, glycopeptides, tigecycline, daptomycin, linezolid, tetracyclines, vancomycin, penicillins + β-lactamase inhibitors.

### Data collection

All available data were collected from the electronic medical records of HTD, including basic demographic characteristics (i.e. age, sex, admission process), clinical metadata (*i.e.*, comorbidities, Intensive Care Unit (ICU) stay, length of hospital stay, discharge outcome), treatment data (i.e., antibiotics, other medications, oxygen therapy, Extracorporeal Membrane Oxygenation (ECMO), hemodialysis, invasive procedures) and microbiological data (i.e., pathogens, dates of sample collection and positive culture, place of sample collection, clinical diagnosis, antimicrobial susceptibility results).

### Statistical analysis

Descriptive statistics were entered in the form of median and proportion. Continuous variables were presented as median (interquartile range, IQR), while categorical variables were summarized with frequencies and percentages. The univariate logistic regression model was conducted to assess the association of mortality and the following variables: age, gender, comorbidities, supplemental oxygen, other invasive procedures, length of hospital stays, and microbiological culture results. The odds ratio (OR) and its corresponding 95% confidence intervals (CI) were calculated to estimate the effect size of each variable. A multivariate logistic regression model included variables significantly associated with mortality from univariate analyses (*p* < 0.05). Interval-censored time to survival was compared between groups using a lognormal accelerated failure time regression model. The distribution of time to survival was visualized using the Kaplan - Meier curve. A *p-*value ≤ 0.05 was considered statistically significant. All data analyses were performed using R Studio version 4.3.0.

## Results

### Demographic characteristics of hospitalized COVID-19 patients

**Fig. 1:**
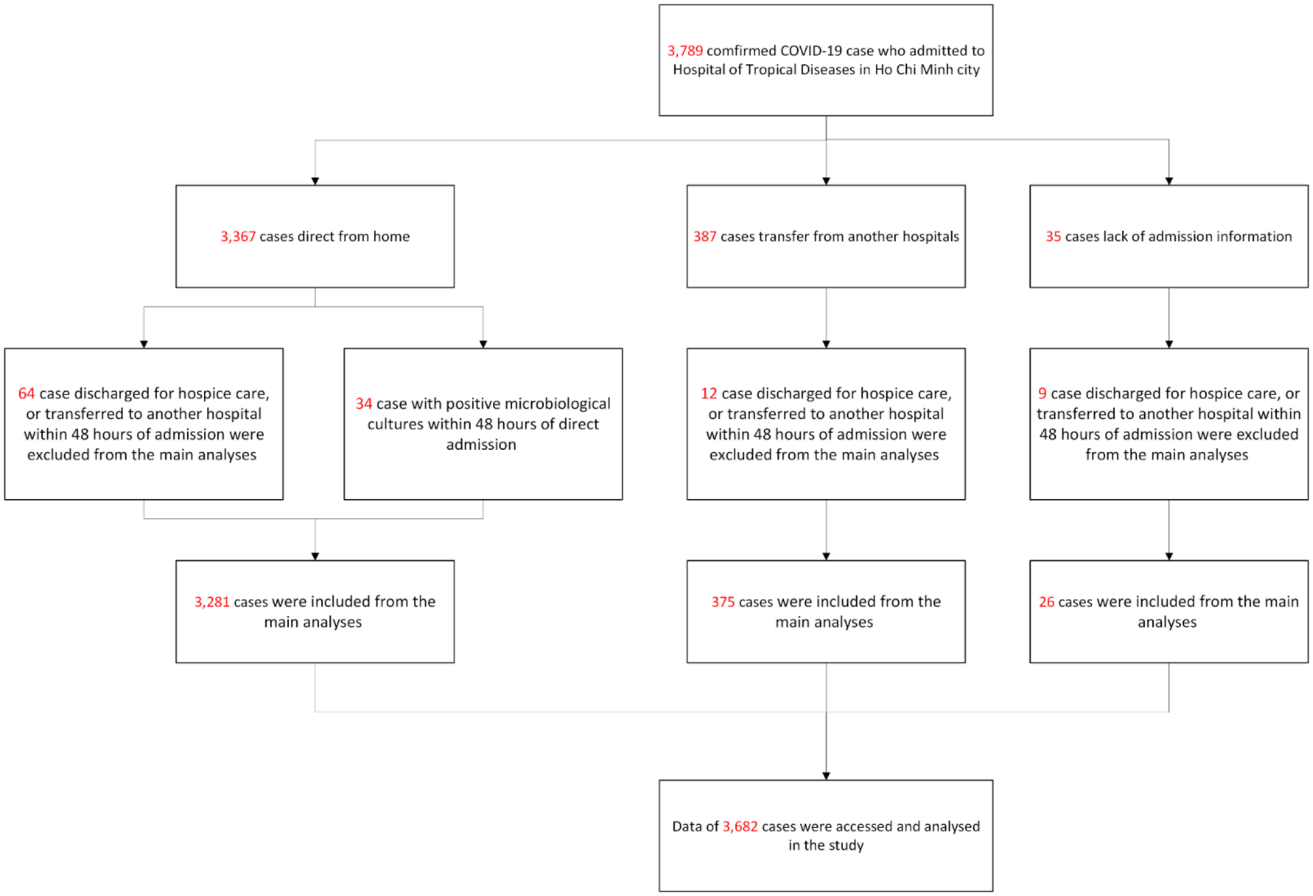
Flow chart of the study

Between 2020 and 2021, a total of 3,789 COVID-19 patients were admitted to the hospital for inpatient care. In this study, we focused on secondary infection that occurred after 48 h of direct admission or within seven days of transfer from another treatment facility. Consequently, patients with positive microbiological cultures within 48 h of direct admission (*n* = 34), discharged for hospice care, or transferred to another hospital within 48 h of admission (*n* = 73) were excluded from the main analyses (Fig. 1). Among 3,682 patients included in the final dataset, 375 (10.3%) were transferred to HTD from quarantine areas or another healthcare facilities. The prevalence of microbiologically confirmed secondary infection was 17.7% (651/3,682), while suspected secondary infection accounted for 16.6% (613/3,682) of patients and 65.5% were classified as having no secondary infection (Table 1).

The median age of patients was 54 years (IQR, 39–65). Those with microbiologically confirmed secondary infections were older, with a median age of 63 years (IQR, 54–72), compared to 49 years (IQR, 34–61) in the no secondary infection group. Males accounted for 44% (1,621/3,682) of patients, with a similar distribution observed across the three patient groups. The most common comorbidities observed in the hospitalized COVID-19 patients were cardiovascular diseases (CVD) (74.9%, 2,757/3,682), hypertension (65.9%, 2,428/3,682), diabetes (54.5%, 2,005/3,682) and chronic kidney diseases (14.4%, 529/3,682). The prevalence of asthma, cancer, chronic obstructive pulmonary disease (COPD), acquired immune deficiency syndrome (AIDS), and obesity varied between 2.9% and 6.4% (Table 1).

**Table 1:** Demographic characteristics of COVID-19 patients

	Overall, *N* = 3,682 (100%)^1^	No secondary infection, *N* = 2,418 (65.6%)^1^	Suspected secondary infection, *N* = 613 (16.6%)^1^	Microbiologically confirmed secondary infection, *N* = 651 (17.7%)^1^
**Demographics**				
** Age (years)**	54 (39, 65)	49 (34, 61)	60 (49, 72)	63 (54, 72)
** Age group (years)**				
0–18	170 (4.6%)	163 (6.7%)	5 (0.8%)	2 (0.3%)
19–34	561 (15.2%)	469 (19.4%)	56 (9.1%)	36 (5.5%)
35–44	501 (13.6%)	394 (16.3%)	57 (9.3%)	50 (7.7%)
45–54	709 (19.3%)	499 (20.6%)	115 (18.8%)	95 (14.6%)
55–64	807 (21.9%)	456 (18.9%)	153 (25%)	193 (30.4%)
65+	934 (25.4%)	437 (18.1%)	227 (37%)	270 (41.5%)
** Sex**,** male**	1,621 (44%)	1,087 (45%)	272 (44.4%)	262 (40.2%)
**Comorbidities**				
Asthma	115 (3.1%)	63 (2.6%)	26 (4.2%)	26 (4%)
Cancer	123 (3.3%)	66 (2.7%)	28 (4.6%)	29 (4.5%)
Cardiovascular diseases	2,757 (74.9%)	1,651 (68.3%)	515 (84%)	591 (90.8%)
Chronic kidney diseases	529 (14.4%)	251 (10.4%)	132 (21.5%)	146 (22.4%)
COPD	108 (2.9%)	58 (2.4%)	26 (4.2%)	24 (3.7%)
Diabetes	2,005 (54.5%)	1,125 (46.5%)	365 (59.4%)	515 (79.1%)
AIDS	118 (3.2%)	69 (2.9%)	21 (3.4%)	28 (4.3%)
Hypertension	2,428 (65.9%)	1,410 (58.3%)	468 (76.3%)	550 (84.5%)
Obesity	237 (6.4%)	136 (5.6%)	34 (5.5%)	67 (10.3%)

^1^Values are presented as numbers (%) or median (interquartile range) or n (%), and proportions (%) are calculated based on column totals. COPD: chronic obstructive pulmonary disease, AIDS: acquired immunodeficiency syndrome, ECMO: extracorporeal membrane oxygenation

### Treatment characteristics of hospitalized COVID-19 patients

Of 3,682 patients, 2,138 (58.1%) received non-invasive oxygen therapies (face mask, nasal cannula, noninvasive positive pressure ventilation (NIPPV), and high flow nasal cannula), whereas 747 (20.3%) required mechanical ventilation. The use of mechanical ventilation was higher in patients with microbiologically confirmed secondary infection (88.2%, 574/651) compared to those with suspected (19.1%, 117/613) and those with no secondary infection (2.3%, 56/2,418). Similarly, patients with microbiologically confirmed secondary infection experienced a higher frequency of hemodialysis and ECMO (23% and 5.2%) compared to the suspected (3.6% and 0%) and no secondary infection (0.1% and 0%) groups, respectively (Table 2).

There were 3,506 patients (95.2%) receiving at least one course of medication during hospitalization, among which antibiotics were most common (73,1%), followed by antithrombotic (72.6%), immunosuppressant (68.7%), antifungal (12.9%) and antiviral drugs (8.4%). Of note, almost all patients with microbiologically confirmed (median duration: 20 days) and suspected secondary infection (median duration: 13 days) were prescribed antibiotics, while 59.2% (1,431/2418) of patients without secondary infection also received antibiotics for a median length of 8 days. Additionally, the frequency of antifungal use was significantly higher in patients with microbiologically confirmed (64.8%, 422/651) compared to those with suspected (6.2%, 38/613) and no secondary infection (0.6%, 15/2,242) (Table 2).

**Table 2:** Treatment characteristics of COVID-19 patients

	Overall, *N* = 3,682 (100%)^1^	No secondary infection, *N* = 2,418 (65.6%)^1^	Suspected secondary infection, *N* = 613 (16.6%)^1^	Microbiologically confirmed Secondary infection, *N* = 651 (17.7%)^1^
Supplemental oxygen requirement				
Noninvasive ventilation	2,138 (58.1%)	1,043 (43.1%)	516 (84.2%)	579 (88.9%)
Mechanical ventilation	747 (20.3%)	56 (2.3%)	117 (19.1%)	574 (88.2%)
**Other Invasive procedures**				
ECMO	34 (0.9%)	0 (0%)	0 (0%)	34 (5.2%)
Hemodialysis	175 (4.8%)	3 (0.1%)	22 (3.6%)	150 (23%)
**Medicinal Treatment**	3,506.0 (95.2%)	2,242.0 (92.7%)	613.0 (100%)	651.0 (100%)
Antibiotic	2,692.0 (73.1%)	1,431.0 (59.2%)	610.0 (99.5%)	651.0 (100%)
Antiviral	308.0 (8.4%)	166.0 (6.9%)	91.0 (14.8%)	51.0 (7.8%)
Antifungal	475.0 (12.9%)	15.0 (0.6%)	38.0 (6.2%)	422.0 (64.8%)
Immunosuppressant	2,528.0 (68.7%)	1,297.0 (53.6%)	592.0 (96.6%)	639.0 (98.2%)
Antithrombotic	2,672.0 (72.6%)	1,420.0 (58.7%)	606.0 (98.9%)	646.0 (99.2%)
**Duration (days)**,** median (IQR)**				
Antibiotic	11 (8, 16)	8 (7, 11)	13 (10, 17)	20 (14, 31)
Antiviral	5 (4, 5)	5 (4, 5)	5 (5, 5)	5 (4, 5)
Antifungal	9 (5, 14)	7 (5, 10)	7 (3, 10)	10 (5, 14)
Immunosuppressant	9 (7, 11)	8 (7, 10)	9 (7, 11)	10 (9, 14)
Antithrombotic	12 (8, 16)	10 (8, 13)	13 (9, 18)	17 (11, 27)
**Length of hospital stay (days)**,** median (IQR)**	14 (10, 19)	13 (9, 16)	17 (13, 22)	21 (14, 35)
**Outcome**,** Survival**	3,075 (83.5%)	2,351 (97.2%)	492 (80.3%)	232 (35.6%)

^1^n (%), Proportions (%) are calculated based on column totals

### Clinical outcomes of hospitalized COVID-19 patients

**Fig. 2:**
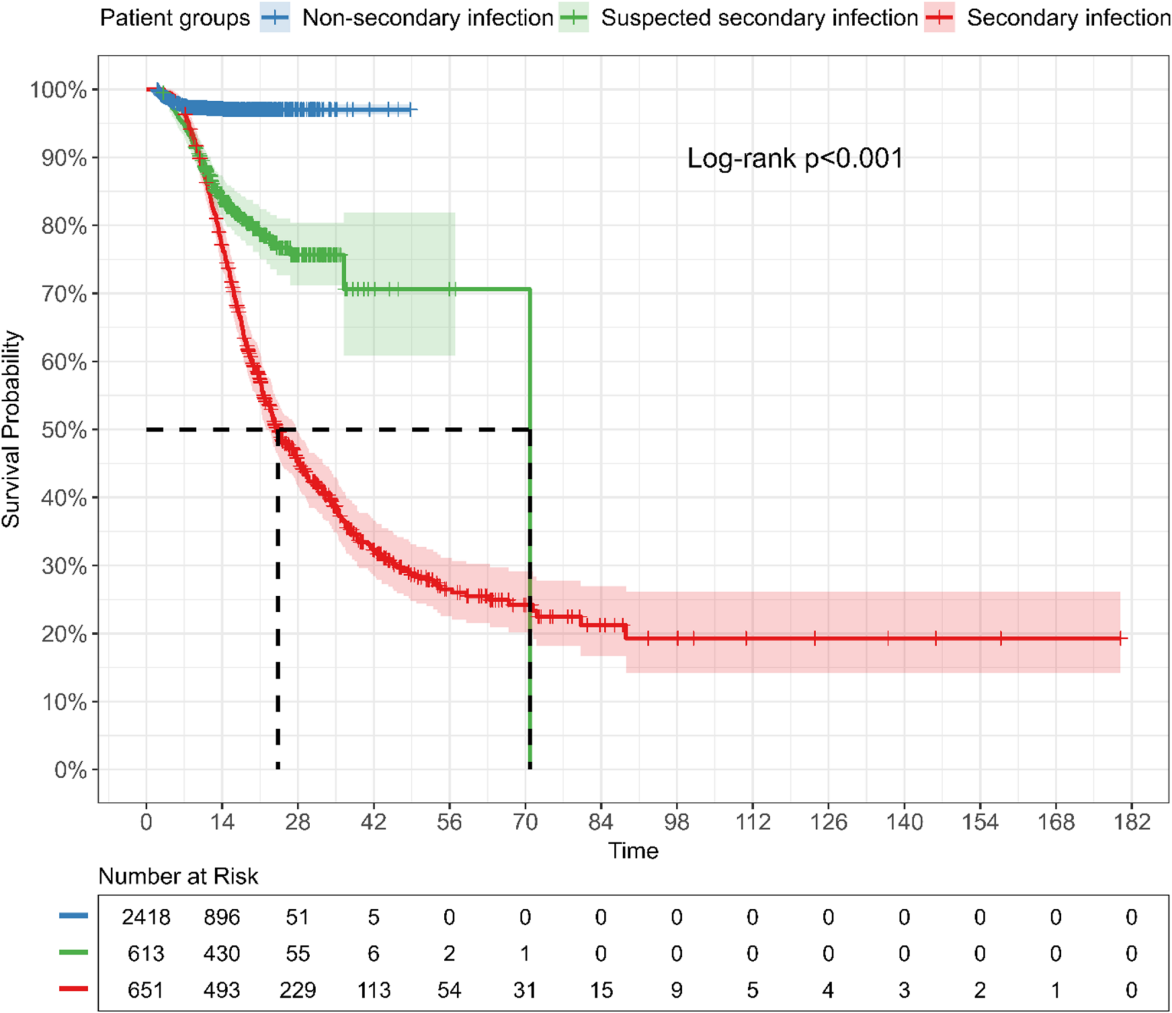
Kaplan-Meier survival estimates by patient groups. Kaplan-Meier survival curves comparing patient groups. The x-axis represents time (in days), and the y-axis represents the probability of survival. Distinct colors or line types indicate different patient groups. Vertical ticks on the curves mark censored data points. Statistical significance between survival curves was evaluated using the log-rank test

The overall survival rate was 83.5% (3,075/3,682) (Table 2). Notably, the survival outcome was highest in patients without secondary infection (97.2%, 2,351/2,418), followed by patients with suspected secondary infection (80.3%, 492/613) and dropped dramatically to 35.6% (232/651) in those with microbiologically confirmed secondary infection. The mean length of hospital stay was 14 days (IQR, 10–19). Patients with microbiologically confirmed secondary infections had a longer median hospital stay (21 days) compared to those with suspected (17 days) and no secondary infections (13 days). Additionally, the Kaplan–Meier survival curve indicates that the survival probability for patients with microbiologically confirmed secondary infection was 50% on day 25 of hospital admission and declined to 28.3% on day 50 (Fig. 2), significantly lower than those observed in the suspected (76.7% on day 25 and 70.6% on day 50) and no secondary infection groups (97% on both days) (log-rank test, *p* < 0.001).

### Distribution of pathogens and source of isolation

A total of 2,649 non-duplicate pathogens were identified from microbiological culture, including 1,343 (50.7%) from respiratory samples, 800 (30.2%) from urine, 413 (15.6%) from blood, and 93 (3.5%) from other samples. Gram-negative bacteria were predominant, comprising 53.8% (1,425/2,649) of identified organisms, followed by fungal pathogens (32.6%, 861/2,649) and Gram-positive bacteria (13.7%, 363/2,649).

The predominant Gram-negative pathogens were *A. baumannii* (16.6%), *K. pneumoniae* (12.6%), and *P. aeruginosa* (9.9%). Among fungal pathogens, the most prevalent were *Candida tropicalis* (13.4%), *Candida albicans* (9.9%), and *Candida glabrata* (3.4%). *Enterococcus faecium* (7.5%), *Staphylococcus aureus* (2.1%), and *Enterococcus faecalis* (1.2%) were the most common Gram-positive pathogens (Table 3).

Gram-negative pathogens were predominantly found in lower respiratory samples, with *(A) baumannii* being the most common (27.9%, 376/1,343), followed by *K. pneumoniae* (15.6%, 210/1,343), *P. aeruginosa* (15.7%, 211/1,343), *(B) cepacia* (6.5%, 87/1,3343) and *S. maltophilia* (4.5%, 60/1,343). Fungal pathogens were over-represented in urine samples, with the dominance of *(C) tropicalis* (31.9%, 255/800), *C. albicans* (16.7%, 134/800), and *C. glabrata* (9.3%, 74/800). The predominant bacteria found in urine were *E. faecium* (19%, 152/800) and *K. pneunoniae* (6.1%, 49/800). The two Gram-negative pathogens, *K. pneumoniae* (16.7%, 69/413) and *A. baumannii* (12.8%, 53/413) were also prevalent in blood samples, followed by the Gram-positive *Enterococcus faecium* (8%, 33/413) and the fungal pathogen *Candida tropicalis* (7%, 29/413) (Table 3).

**Table 3:** Distribution of pathogens by source of isolation

Pathogen	Overall *N* = 2,649 (100%)^*1*^	Blood *N* = 413 (15.6%)^*1*^	Lower respiratory tract *N* = 1,343 (50.7%)^*1*^	Urine *N* = 800 (30.2%)^*1*^	Other samples *N* = 93 (3.5%)^*1*^
**Gram-negative**					
* Acinetobacter baumannii*	440 (16.6%)	53 (12.8%)	376 (28%)	5 (0.6%)	6 (6.5%)
* Klebsiella pneumoniae*	334 (12.6%)	69 (16.7%)	210 (15.6%)	49 (6.1%)	6 (6.5%)
* Pseudomonas aeruginosa*	262 (9.9%)	17 (4.1%)	211 (15.7%)	16 (2.0%)	18 (19.4%)
* Burkholderia cepacia*	94 (3.5%)	7 (1.7%)	87 (6.5%)	0 (0%)	0 (0%)
* E. meningoseptica*	83 (3.1%)	3 (0.7%)	80 (6.0%)	0 (0%)	0 (0%)
* S. maltophilia*	83 (3.1%)	23 (5.6%)	60 (4.5%)	0 (0%)	0 (0%)
* Escherichia coli*	32 (1.2%)	4 (1%)	7 (0.5%)	21 (2.6%)	0 (0%)
Others	97 (3.7%)	22 (5.3%)	60 (4.5%)	12 (1.5%)	3 (3.2%)
**Gram-positive**					
* Enterococcus faecium*	197 (7.4%)	33 (8%)	6 (0.4%)	152 (19%)	6 (6.5%)
* Staphylococcus aureus*	57 (2.1%)	13 (3.1%)	36 (2.7%)	1 (0.1%)	7 (7.5%)
* Enterococcus faecalis*	33 (1.2%)	24 (5.8%)	4 (0.3%)	5 (0.6%)	0 (0%)
* Staphylococcus haemolyticus*	32 (1.2%)	32 (7.7%)	0 (0%)	0 (0%)	0 (0%)
* Staphylococcus hominis*	12 (0.5%)	11 (2.7%)	1 (0.1%)	0 (0%)	0 (0%)
* Corynebacterium striatum*	6 (0.2%)	0 (0%)	6 (0.4%)	0 (0%)	0 (0%)
* Streptococcus pneumoniae*	4 (0.2%)	2 (0.5%)	2 (0.1%)	0 (0%)	0 (0%)
Others	22 (0.8%)	17 (4.1%)	2 (0.1%)	3 (0.4%)	0 (0%)
**Fungi**					
* Candida tropicalis*	354 (13.4%)	29 (7%)	58 (4.3%)	255 (31.9%)	12 (12.9%)
* Candida albicans*	263 (9.9%)	24 (5.8%)	83 (6.2%)	134 (16.8%)	22 (23.7%)
* Candida glabrata*	91 (3.4%)	2 (0.5%)	13 (1.0%)	74 (9.3%)	2 (2.2%)
* Trichosporon asahii*	36 (1.4%)	3 (0.7%)	0 (0%)	31 (3.9%)	2 (2.2%)
* Candida orthopsilosis*	29 (1.1%)	12 (2.9%)	4 (0.3%)	13 (1.6%)	0 (0%)
* Candida parapsilosis*	21 (0.8%)	6 (1.5%)	4 (0.3%)	11 (1.4%)	0 (0%)
* Candida dubliniensis*	16 (0.6%)	1 (0.2%)	13 (1.0%)	2 (0.3%)	0 (0%)
Others	51 (1.9%)	6 (1.5%)	10 (1.5%)	16 (2.0%)	9 (9.7%)

^1^n (%), Proportions (%) are calculated based on column totals

### Antimicrobial resistance profiles of predominant pathogens in secondary infection

The proportion of resistance to commonly used antibiotics was notably high among the predominant Gram-negative pathogens. Among the tested *A. baumannii* isolates, 29% (108/372) were classified as MDR and 67.5% (251/372) as XDR. The resistance levels were extremely high for carbapenems (95.2%, 354/372), 3rd /4th generation cephalosporins (96%, 357/372), fluoroquinolones (97%, 360/371), penicillins + β-lactamase inhibitors (98.9%, 367/371) and aminoglycosides (87.3%, 324/371). Similarly, 96% (263/274) of the tested *K. pneumoniae* isolates were MDR, with high levels of resistance observed for carbapenems (81.5%, 224/275), penicillins + βa-lactamase inhibitors (92.7%, 255/275), fluoroquinolones (94.5%, 260/275), 3rd /4th -generation cephalosporins (89.5%, 246/275). For *P. aeruginosa* isolates, 65.3% (145/222) were identified as MDR and 8.1% (18/222) as XDR. The proportion of resistance was 79.3% for carbapenems, 84.2% for 3rd /4th -generation cephalosporins, 73.9% for fluoroquinolones, 74.5% for penicillins + β-lactamase inhibitors, and 64.4% for aminoglycosides. Colistin resistance was observed in 37% (101/273) of *K. pneumoniae*, 9.9% (22/222) of *P. aeruginosa*, and 8.4% (31/371) of *A. baumannii* isolates. Fosfomycin resistance also reached 17.9% (37/207) among the tested *K. pneumoniae* isolates (Table 4).

Among the Gram-positive bacteria, 93.5% (43/46) of *S. aureus* and 67.1% (116/173) of *E. faecium*, and 5.3% (1/19) of *E. faecalis* isolates were MDR (Table 4). *S. aureus* isolates displayed a high frequency of resistance to oxacillin (91.3%, 42/46), macrolides/lincosamides (95.7%, 44/46), fluoroquinolones (84.4%), but were susceptible to vancomycin, teicoplanin, and linezolid. For *E. faecium* isolates, the resistance rate was 98.8% for penicillins (ampicillin and benzylpenicillin), 94.7% for erythromycin, 98.8% for fluoroquinolones, 100% for daptomycin, 57.2% for glycopeptides (vancomycin and teicoplanin), 26% for tetracyclines and 1.8% for linezolid. Among the tested *E. faecalis* isolates, a high resistance rate was observed for tetracyclines (89.5%, 17/19), erythromycin (63.2%, 12/19), and fluoroquinolones (57.9% (11/19). However, resistance to ampicillin, benzylpenicillin, vancomycin, teicoplanin, and linezolid was less than 10%. Among the predominant fungal pathogens, 91.2% (31/34) of *C. glabrata* and 44.3% (109/246) of *C. tropicalis* isolates showed resistance to fluconazole, the first-line antifungal drug. These organisms also exhibited resistance to voriconazole, with a prevalence of 28.6% for *C. glabrata* and 21.4% for *C. tropicalis*. In contrast, only 6.1% (13/213) of *C. albicans* were resistant to fluconazole. Resistance to echinocandins (caspofungin, micafungin) and amphotericin B was low across the three dominant fungal pathogens, ranging between 0.4% and 8.6% (Table 4).

**Table 4:** The distribution of antimicrobial resistance in predominant bacterial and fungal pathogens in secondary infections

	**Gram-negative pathogens**		
	Acinetobacter baumannii (*N* = 440)	Klebsiella pneumoniae (*N* = 334)	Pseudomonas aeruginosa (*N* = 262)
**Drug-resistant**			
Multidrug-resistant (MDR)	29% (108/372)	96.0% (263/274)	65.3% (145/222)
Extensively drug-resistant (XDR)	67.5% (251/372)	0.0% (0/274)	8.1% (18/222)
**Antimicrobial categories** ^1^			
Aminoglycosides	87.3% (324/371)	81.1% (223/275)	64.4% (143/222)
Penicillins + β-lactamase inhibitors	98.9% (367/371)	92.7% (255/275)	74.5% (166/222)
Carbapenems	95.2% (354/372)	81.5% (224/275)	79.3% (176/222)
Cephalosporins (3rd /4th)	96% (357/372)	89.5% (246/275)	84.2% (187/222)
Fosfomycin	20.0% (1/5)	17.9% (37/207)	-
Polymyxins (colistin)	8.4% (31/371)	37% (101/273)	9.9% (22/222)
Fluoroquinolones	97% (360/371)	94.5% (260/275)	73.9% (164/222)
Tetracyclines	50% (3/6)	42.7% (70/164)	-
Trimethoprim/sulfamethoxazole	78.7% (292/371)	61.4% (167/272)	-
	**Gram-positive pathogens**		
	Enterococcus faecium (*N* = 197)	Staphylococcus aureus (*N* = 57)	Enterococcus faecalis (*N* = 33)
**Drug-resistant**			
Multi-drug-resistant (MDR)	67.1% (116/173)	93.5% (43/46)	5.3% (1/19)
Extensively drug-resistant (XDR)	0.0% (0/173)	0.0% (0/46)	0.0% (0/19)
**Antimicrobial Categories** ^1^			
Aminoglycosides (gentamicin)	-	66.7% (30/45)	-
Penicillins	98.8% (171/173)	95.7% (44/46)	10.5% (2/19)
Glycopeptides	57.2% (99/173)	0.0% (0/46)	5.3% (1/19)
Macrolides/lincosamides	94.7% (161/170)	95.7% (44/46)	63.2% (12/19)
Lipopeptides (daptomycin)	100.0% (39/39)	-	-
Oxazolidinones (linezolid)	1.8% (3/171)	0.0% (0/45)	5.6% (1/18)
Fluoroquinolones	98.8% (171/173)	84.4% (38/45)	57.9% (11/19)
Tetracyclines	26% (45/173)	0.0% (0/45)	89.5% (17/19)
	**Fungal pathogens**		
	Candida tropicalis (*N* = 355)	Candida albicans (*N* = 263)	Candida glabrata (*N* = 91)
Flucytosine^1^	0.4% (1/256)	1.4% (3/213)	1.6% (1/63)
Amphotericin B^1^	0.4% (1/259)	0.5% (1/214)	0.0% (0/63)
Caspofungin^1^	1.5% (4/260)	2.3% (5/214)	8.6% (5/58)
Fluconazole^1^	44.3% (109/246)	6.1% (13/213)	91.2% (31/34)
Micafungin^1^	1.2% (3/260)	1.9% (4/211)	1.6% (1/62)
Voriconazole^1^	21.4% (53/248)	1.4% (3/211)	28.6% (8/28)

^1^% resistance (number of resistant isolates / total number of isolates tested)

### Factors associated with mortality in hospitalized COVID-19 patients

According to univariate logistic regression analyses, patients with suspected (OR: 8.63, *p* < 0.001) or microbiologically confirmed secondary infection (OR: 63.4, *p* < 0.001) had significantly higher odds of mortality, compared to those with no secondary infection (reference group). Older age (35–44 years, OR: 10, *p* = 0.024; 45–54 years, OR: 18.5, *p* = 0.004; 55–64 years, OR: 43.1, *p* < 0.001; 65+, OR: 93.2, *p* < 0.001) was also associated with increased mortality compared to the 0–18 years age group (reference group). Comorbidities such as cancer (OR: 2.08, *p* < 0.001), cardiovascular disease (OR: 6.45, *p* < 0.001), chronic kidney disease (OR: 2.91, *p* < 0.001), COPD (OR: 1.72, *p* = 0.017), diabetes (OR: 4.21, *p* < 0.001), and hypertension (OR: 3.94, *p* < 0.001), as well as the use of oxygen therapy (non-invasive: OR: 4.94, *p* < 0.001; mechanical ventilation: OR: 109, *p* < 0.001) and invasive procedures like hemodialysis (OR: 16.2, *p* < 0.001), were significantly linked to higher mortality.

A multivariate logistic regression model incorporated significant predictors from the univariate analyses. Multivariate analysis showed that suspected (OR: 2.75, *p* < 0.001) and microbiologically confirmed secondary infection (OR: 2.22, *p* = 0.001) were independently associated with higher mortality compared to those with no secondary infection. Compared to the 0–18 years age group, older age (55–64 years, OR: 9.14, *p* = 0.046; 65+, OR: 32.4, *p* = 0.002) was also an independent predictor of mortality, as were chronic kidney disease (OR: 1.69, *p* = 0.005), cardiovascular disease (OR: 2.54, *p* < 0.001) and mechanical ventilation (OR: 79.9, *p* < 0.001) (Table 5).

**Table 5:** Prediction factors associated with mortality in COVID-19 patients

Characteristic	Univariate			Multivariable		*p*-value
	OR	95% CI	*p*-value	OR	95% CI	
Secondary infection group						
No secondary infection	1.00			1.00		
Suspected secondary infection	8.63	6.33, 11.9	**< 0.001**	2.75	1.76, 4.31	**< 0.001**
Microbiologically confirmed secondary infection	63.4	47.7, 85.4	**< 0.001**	2.22	1.36, 3.60	**0.001**
**Demographics**						
** Age group (years)**						
0–18	1.00			1.00		
19–34	3.69	0.72, 67.5	0.2	1.64	0.24, 33.5	0.7
35–44	10.0	2.11, 179	**0.024**	2.89	0.45, 57.6	0.3
45–54	18.5	4.06, 328	**0.004**	4.94	0.80, 97.1	0.2
55–64	43.1	9.57, 761	**< 0.001**	9.14	1.50, 179	**0.046**
65+	93.2	20.8, 1,642	**< 0.001**	32.4	5.34, 633	**0.002**
** Sex**,** male**	0.80	0.67, 0.96	**0.016**	1.08	0.79, 1.46	0.6
**Length of stay (days)**	1.00	0.99, 1.01	> 0.9			
**Comorbidity**						
Asthma	1.28	0.78, 2.00	0.3	—	—	—
Cancers	2.08	1.37, 3.08	**< 0.001**	1.84	0.92, 3.65	0.082
Cardiovascular diseases	6.45	4.63, 9.27	**< 0.001**	2.54	1.53, 4.30	**< 0.001**
Chronic kidney diseases	2.91	2.36, 3.58	**< 0.001**	1.69	1.16, 2.45	**0.005**
COPD	1.72	1.08, 2.656	**0.017**	0.67	0.30, 1.48	0.3
Diabetes	4.21	3.42, 5.23	**< 0.001**	1.23	0.87, 1.75	0.2
AIDS	1.31	0.81, 2.03	0.2			
Hypertension	3.94	3.11, 5.06	**< 0.001**	0.95	0.63, 1.43	0.8
Obesity	1.13	0.79, 1.58	0.5			
**Supplemental oxygen**						
Non-invasive	4.94	3.94, 6.26	**< 0.001**	0.67	0.44, 1.01	**0.056**
Mechanical ventilation	109	82.4, 147	**< 0.001**	79.9	51.4, 127	**< 0.001**
**Invasive procedure**						
ECMO	1.84	0.81, 3.81	0.12	—	—	—
Hemodialysis	16.2	11.5, 23.9	< 0.001	1.28	0.83, 2.02	0.3

OR: Odds Ratio, CI: Confidence Interval, COPD: chronic obstructive pulmonary disease, AIDS: acquired immunodeficiency syndrome, ECMO: extracorporeal membrane oxygenation

## Discussion

We conducted an epidemiological investigation on bacterial and fungal secondary infection in COVID-19 patients hospitalized at a major COVID-19 treatment center in HCMC, Vietnam, between 2020 and 2021. We found a prevalence of 17.7% for microbiologically confirmed secondary infections among the admitted COVID-19 patients. The prevalence of secondary infection has been reported worldwide, varying between 9% and 30%, depending on the country, hospital setting, patient population, and healthcare system capacity [16, 17]. In a recent meta-analysis of nineteen studies, a pooled prevalence of secondary infection in COVID-19 patients was reported at 19% [18]. Consistent with previous reports, we found that the majority of bacterial pathogens originated from lower respiratory samples, while *Candida* spp. predominated urine samples [16, 17, 19]. Furthermore, Gram-negative bacteria such as *A. baumannii*, *K. pneumoniae*, and *P. aeruginosa* were prevalent in both respiratory and blood samples, exhibiting a high frequency of MDR and XDR, including resistance to last-resort antibiotics. Our findings concur with previous publications, highlighting the proliferation of MDR and XDR Gram-negative bacteria during the pandemic [20]. There are imminent threats of establishing endemic circulations of these MDR/XDR strains in hospital settings worldwide after the pandemic, potentially leading to increased demand for last-resort antibiotics and poorer patient outcomes. Furthermore, severe COVID-19 patients are at elevated risk of secondary fungal infection [21]. Here, *C. albicans* and *C. tropicalis* were the most commonly found fungal pathogens, primarily derived from the urinary tract, with a concerning high rate of fluconazole resistance. The World Health Organization (WHO) has recently listed *C. albicans* and *C. tropicalis* as critical and high-risk pathogens, respectively [22]. We need to fill the current gaps in early diagnostics and treatment to improve the clinical outcomes of secondary fungal diseases [23].

Several major contributing factors for secondary infection have been reported, including an overwhelmed healthcare system, compromised hospital infection control and prevention, greater uses of antibiotic and immunosuppressive drugs and invasive procedures, and the endemic circulation of MDR nosocomial pathogens [24–26]. These factors were also observed in our setting and among our study population, in which the hospital faced substantial challenges with the overwhelming numbers of hospitalized COVID-19 patients. Apart from the challenges stemming from the over-burdened healthcare system, we found that almost all COVID-19 patients (73.1%) admitted to our hospital were given broad-spectrum antibiotics, regardless of their severity at presentation. This contrasts with the much lower prevalence of microbiologically-confirmed (17.7%) and suspected secondary infection (16.6%). Our data raise concerns about the overuse of empirical antibiotics aimed at preventing secondary infection, which are common in viral respiratory diseases such as COVID-19. Although directed by a local COVID-19 treatment protocol, the benefits of early treatment of antibiotics are a subject of continuous debate [27–29]. Heavy use of broad-spectrum antibiotics can lead to the selection of MDR and XDR organisms, disruptions of human microbiota, and adverse effects in patients with co-morbidities [25, 30–33]. Together with the common use of immunosuppressive drugs, this creates a favorable condition for the proliferation and spread of nosocomial MDR and XDR bacterial and fungal pathogens. As evidenced in our dataset, patients with microbiologically confirmed secondary infection had a longer duration of antibiotic use, from whom the identified bacterial pathogens (i.e., *A. baumannii*,* K. pneumoniae*,* P. aeruginosa*) often displayed MDR or XDR phenotype. Furthermore, other opportunistic pathogens, including *Burkholderia cepacia*,* Elizabethkingia meningoseptica*,* Stenotrophomonas maltophilia*, and *Candida* spp., were found in these patients. Given that empirical broad-spectrum antibiotics were initiated very early in most patients, with a mean time of two hours from admission and before culture results were available, the causal relationship between antimicrobial use and the occurrence of secondary infection is challenging to determine. Nonetheless, our findings indicate that broad-spectrum antibiotics should not be given as prophylactic therapy without microbiological evidence.

Secondary infection in COVID-19 patients often results in increased mortality compared to those without the infection [29, 34–36]. Here, we also observed a higher mortality rate (64.4%) among patients with microbiologically confirmed secondary infection, compared to 19.7% among suspected secondary infection and 2.8% in patients without secondary infection. This major discrepancy in mortality signifies the established correlation between secondary infection and mortality in COVID-19 patients. Several risk factors of death were identified, including the occurrence of secondary infection, older age, the presence of cardiovascular and kidney disease, and the use of mechanical ventilation, consistent with findings from previous publications [35, 37–41]. The prevention of secondary infection in COVID-19 patients, especially those at higher risk of mortality, should be a priority. This is of particular importance given the fact that many of these patients suffered from multiple infection episodes with highly resistant organisms. During the peak of the COVID-19 pandemic in HCMC, the hospital setting was overloaded and understaffed. Coupled with the insufficient supplies of personal protective equipment and compromised IPC measures, this presented major challenges for preventing secondary infection. This situation underscores the importance of effective hospital IPC and antibiotic stewardship programs, which needs to be strengthened in peace time to address similar devastating scenarios in the future.

Our study has some limitations. Due to the lack of COVID-19 vaccination information, we could not assess the effect of the vaccine on clinical features and the occurrence of secondary infection. Our study was retrospective, and hence, we could not capture all the factors resulting in the development of secondary infection in COVID-19 patients or determine the direct cause of mortality. Another limitation is the potential risk of overadjustment, as mechanical ventilation is both a marker of severe disease and a risk factor for secondary infection. To address this, we performed a sensitivity analysis excluding ventilation, which showed that secondary infection remained significantly associated with mortality, with an increased odds ratio. This supports the robustness of our findings, although the contributions of secondary infection and ventilation remain challenging to disentangle. Although fungal pathogens such as *C. tropicalis*, *C. albicans* and *C. glabrata* are frequently reported in secondary fungal infection among patients with severe COVID-19 ^42–44^, their presence in the respiratory and urine samples may represent colonization. However, in our study, the majority of COVID-19 patients with secondary fungal infection were prescribed antifungals (82%), suggesting a high likelihood of true fungal infections.

## Conclusion

This work underscores a significantly higher mortality in severe COVID-19 patients with secondary infection, compared to those with suspected or no secondary infection in Vietnam. Secondary infection disproportionately affected elderly people with comorbidities and higher use of invasive treatment including mechanical ventilation and hemodialysis. Gram-negative bacterial pathogens were most common, largely found in respiratory samples, while fungal pathogens were frequently detected in urine samples. The prevalence of MDR and XDR bacterial pathogens was exceptionally high, with a notable rise of fungal pathogens. Although, the pandemic has been successfully controlled, the lessons learned from its detrimental impact on the healthcare system remains highly relevant, especially considering its long-term impact of the continued circulation of MDR/XDR bacterial and fungal strains. Our findings highlight the needs to strengthen healthcare system, particularly IPC measures and antibiotic stewardship programs for preventing nosocomial transmission and better preparing for future epidemic situations.

## Data Availability

The data supporting the findings of this study are not openly available due to sensitive reasons. However, they can be obtained from the corresponding author upon reasonable request. The data are stored in controlled access data storage at Oxford University Clinical Research Unit.
